# Supplementary material for: The formation of the ‘footprint of death’ as a mechanism for generating large substrate-bound extracellular vesicles that mark the site of cell death
Source: Nat Commun. 2025 Oct 15;16:9160. doi: 10.1038/s41467-025-64206-3 (PMC12528690; doi:10.1038/s41467-025-64206-3)
Supplement: Supplementary file 1 — Supplementary Information [file 41467_2025_64206_MOESM1_ESM.pdf]

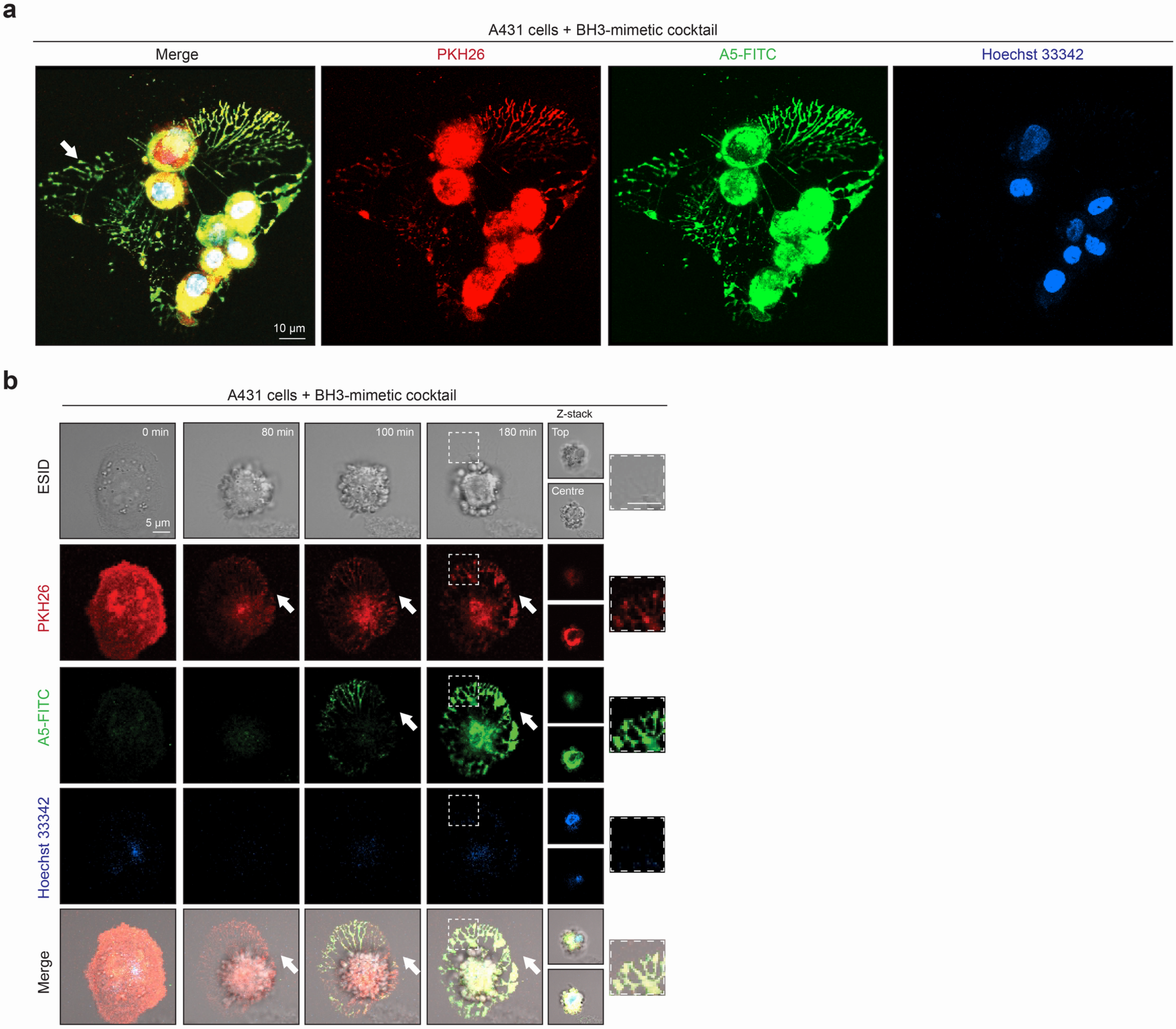

Supplementary Figure 1: Extended time lapse analysis of FOOD generation by A431 epidermal cells.
a,b A431 epidermal cells were treated with a BH3-mimetic cocktail (2.5 μM ABT 737, 0.5 μM S63845) and imaged by time lapse CLSM. Cell membrane and nucleus were visualised by PHK26 (red) and Hoechst 33342 (blue) staining, respectively, and exposed phosphatidyl serine was examined using A5-FITC (green). Cells in **a** are representative of cells 180 min post treatment. Maximum intensity projection (MIP) shown. Time lapse images in **b** show cells at lower focal plane (main), and regions at the top and centre of the cell (right). At least three independent experiments were performed for all experiments unless otherwise specified.

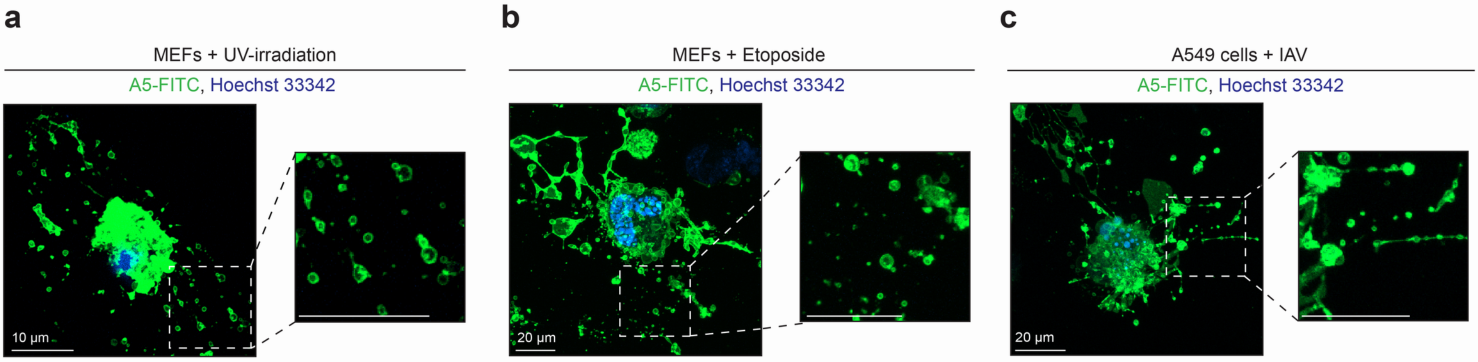

Supplementary Figure 2: FOOD and F-ApoEV formation in response to other methods of apoptosis induction.
Representative MIP images from CLSM of FOOD/F-ApoEV formation in (a) MEFs 24 h post UV-irradiation (150 mJ/cm²), (b) MEFs 24 h post treatment with etoposide (125 nM), and (c) A549 cells 24 post infection with IAV (MOI=10). Enlarged ROI on left. Exposed PtdSer was visualised with A5-FITC and cell nucleus with Hoechst 33342. At least three independent experiments were performed for all experiments unless otherwise specified.

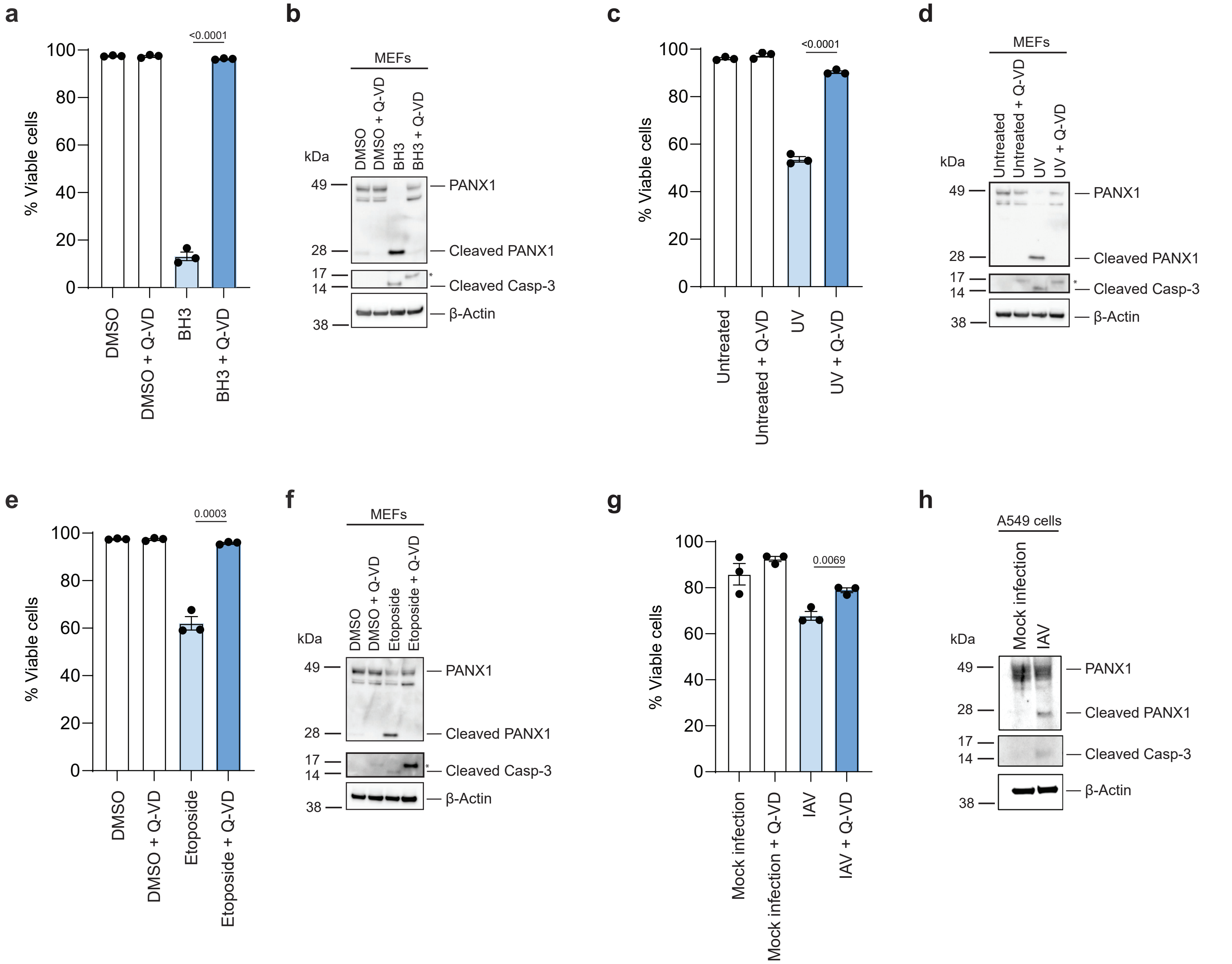

Supplementary Figure 3: Validation of cell death inducing stimuli. The reduction in cell viability was determined by flow cytometry (as measure by phosphatidyl serine exposure using A5-FITC staining) and apoptosis was confirmed by immunoblot analysis of the following: (**a,b**) MEFs 4 h post treatment with BH3-mimetic cocktail (5 μM ABT-737, 10 μM S63845), (**c,d**) MEFs 24 h post UV-irradiation (150 mJ/cm²), (**e,f**) MEFs 24 h post etoposide (125 nM), and (**g,h**) A549 cells 24 h post infection with IAV (MOI=10), with or without the presence of pan-caspase inhibitor Q-VD-OPh (Q-VD) (50 μM) to inhibit apoptosis. Asterisk indicates a non-specific band which occurs due the induction of apoptosis and co-treatment with Q-VD-OPh[72]. Error bars represent s.e.m. At least three independent experiments were performed for all experiments unless otherwise specified.

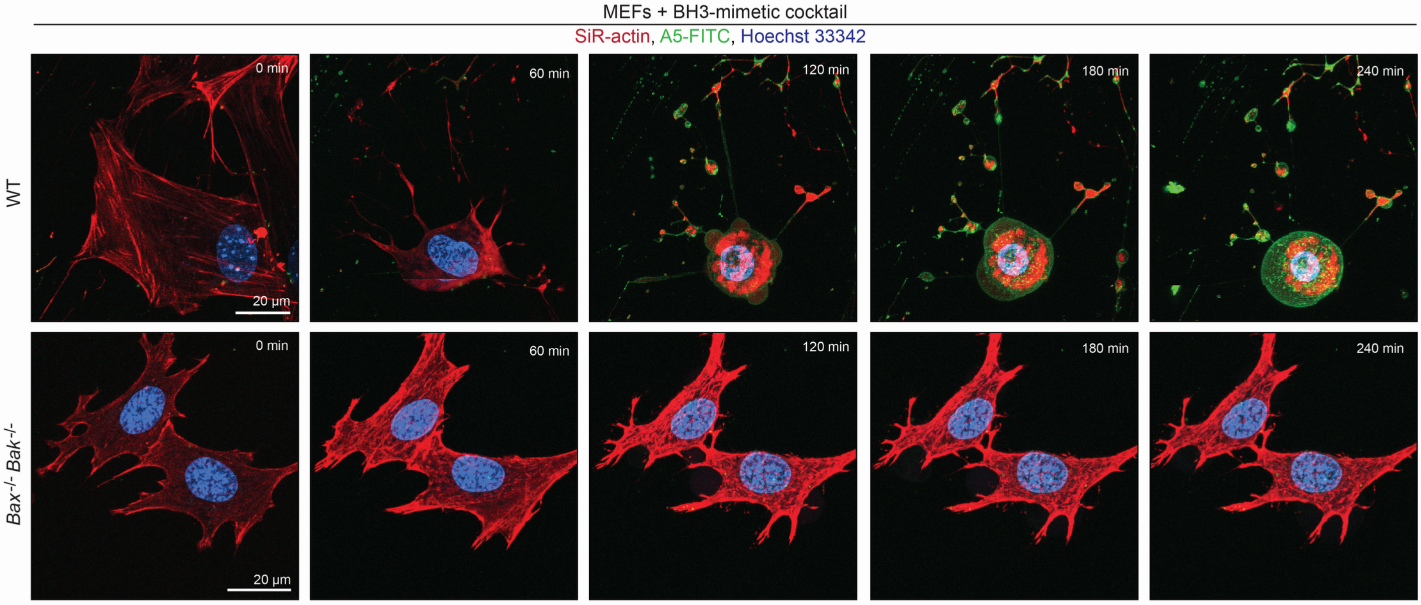

Supplementary Figure 4: The loss of Bax/Bak prevents the formation of FOOD/F-ApoEVs. WT or $Bax^{-/-}$ $Bak^{-/-}$ MEFs were treated with a BH3-mimetic cocktail (5 µM ABT-737, 10 µM S63845) and imaged by time lapse confocal laser scanning microscopy (CLSM) to monitor the formation of FOOD. F-actin and the nucleus were visualised by SiR-actin and Hoechst 33342 staining, respectively, and exposed phosphatidylserine with A5-FITC. At least three independent experiments were performed for all experiments unless otherwise specified.

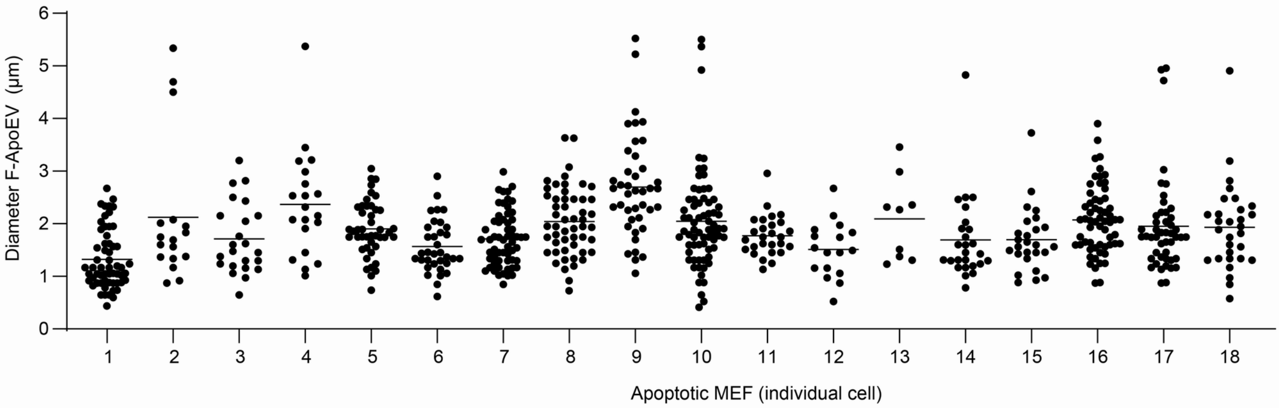

Supplementary Figure 5: Extended data of quantification of the diameter of F-ApoEVs.
Quantification of F-ApoEVs diameter from LLSM imaging, 240 min post apoptosis induction. Data points represent individual F-ApoEVs from (n=18 cells), representative from (n=3) independent experiments.

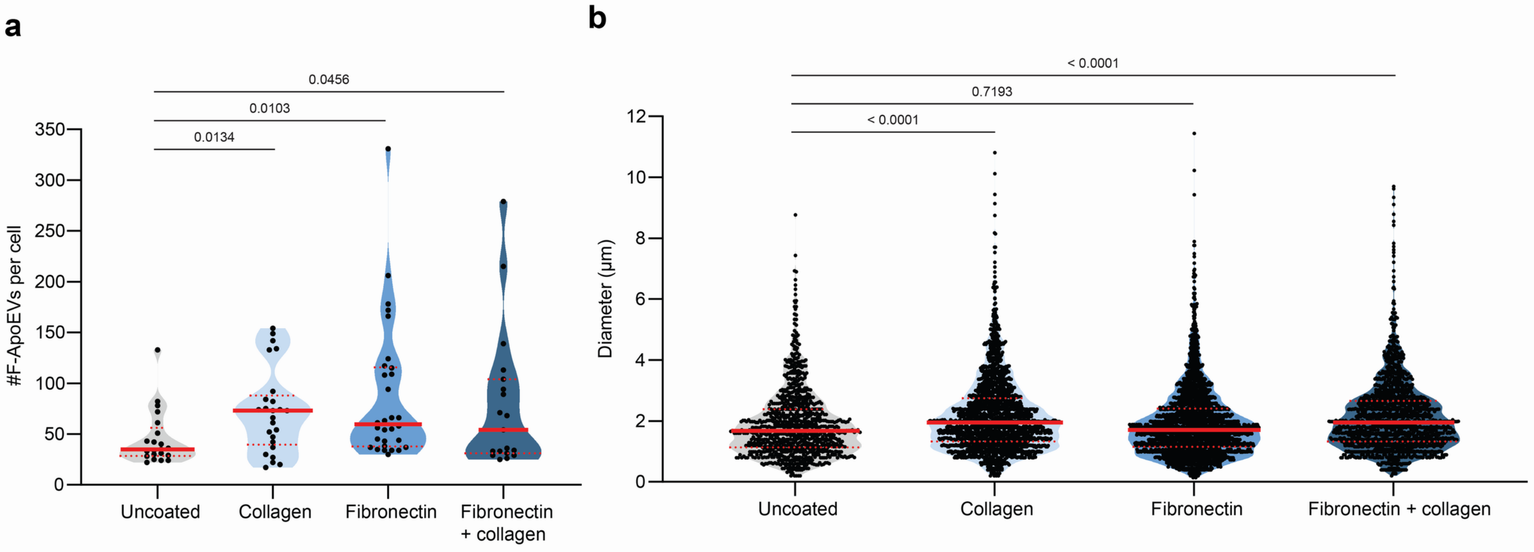

Supplementary Figure 6: FOOD/ F-ApoEVs readily form on surfaces coated with ECM proteins. a Quantification of the number of F-ApoEVs generated per cell from confocal laser scanning microscopy (CLSM) imaging of apoptotic MEFs on uncoated, collagen, fibronectin, and fibronectin and collagen coated chamber slides, treated with a BH3 mimetic cocktail (5 μM ABT-737, 10 μM S63845). Data points represent individual cells (n=21, n=25, n=30 and n=19, respectively) pooled from (n=3) independent experiments. **b** Quantification of F-ApoEV diameter (μm) from CLSM imaging of apoptotic MEFs uncoated, collagen, fibronectin, and fibronectin and collagen coated chamber slides, as previous. Data points represent individual F-ApoEVs (n=952, n=1882, n=2640, and n=1502, respectively) pooled from (n=3) independent experiments. Solid red line indicated mean, dashed red line indicates quarterlies. Unpaired student's two tailed t-test was performed to determine the indicated p-values.

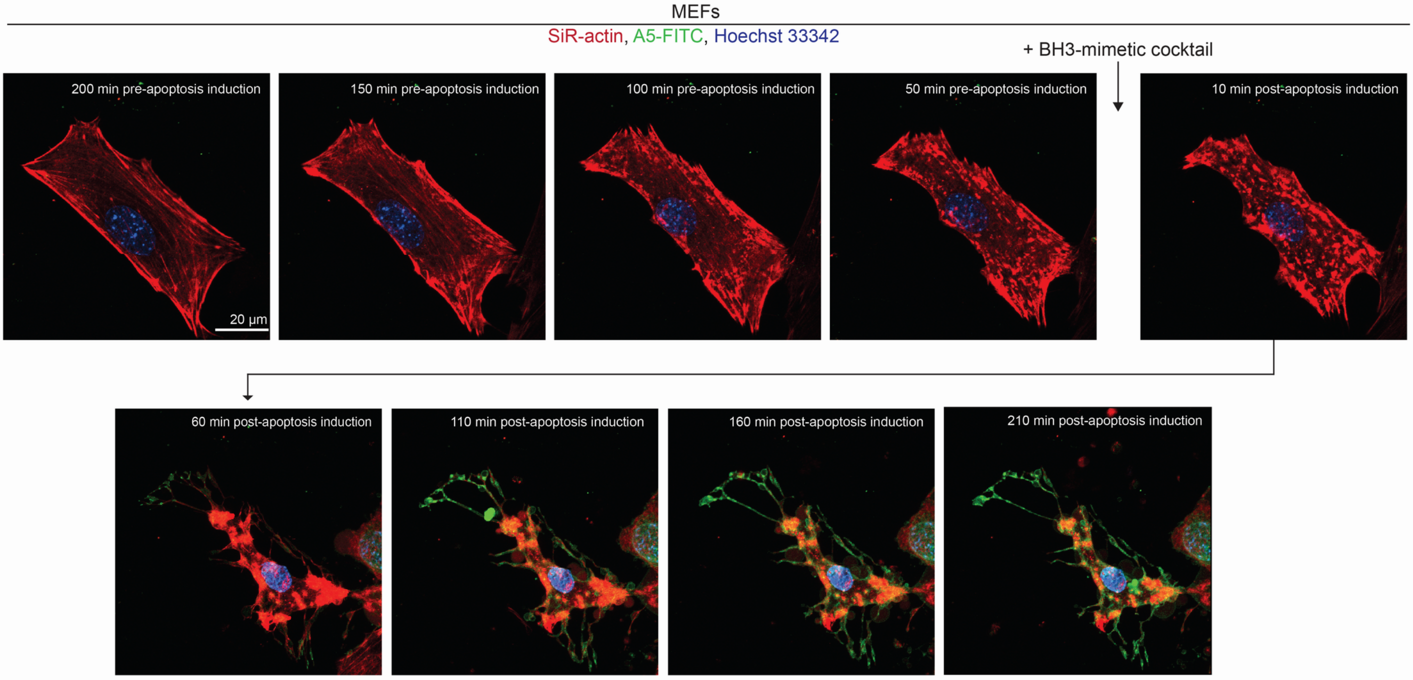

Supplementary Figure 7: FOOD/ F-ApoEV formation does not occur prior to apoptosis induction. MEFs were stained with SiR-actin, A5-FITC, and Hoechst 33342, to visualise F-actin, exposed phosphatidylserine, and nucleus, respectively, and imaged via confocal laser scanning microscopy for 200 min. Imaging was paused and MEFs were then treated with a BH3-mimetic cocktail (5 µM ABT-737, 10 µM S63845) to induce apoptosis, and imaged for an additional 210 min. At least three independent experiments were performed for all experiments unless otherwise specified.

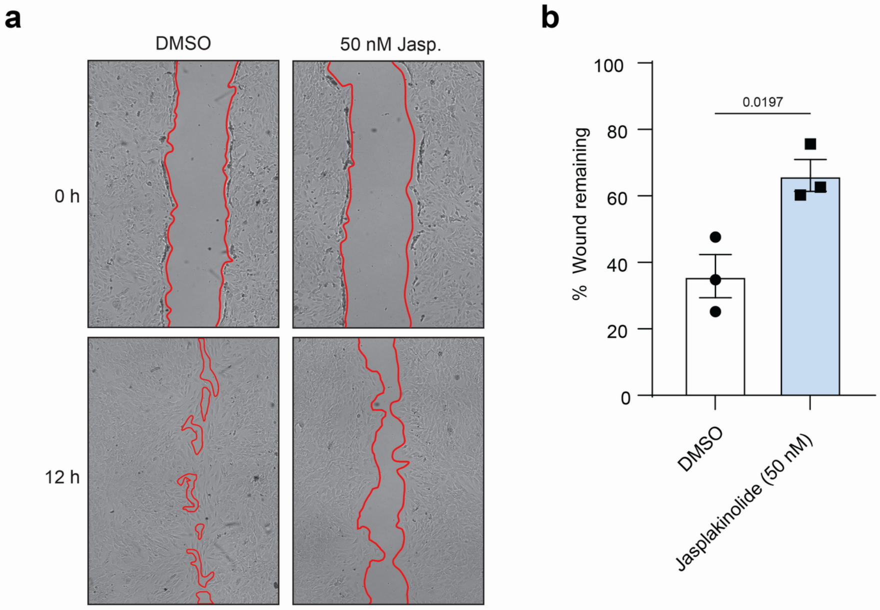

Supplementary Figure 8: Jasplakinolide inhibits cell migration. a Representative bright field images from wound healing migration assay of MEFs 0 h and 12 h post treatment with Jasplakinolide (50 nm) or vehicle control (DMSO). Red line indicates cell boundary.
b Quantification of % wound remaining from bright field images from (a). Data is pooled from (n=3) independent experiments. Error bars represent s.e.m. Unpaired student's two tailed t-test was performed to determine the indicated p-values.

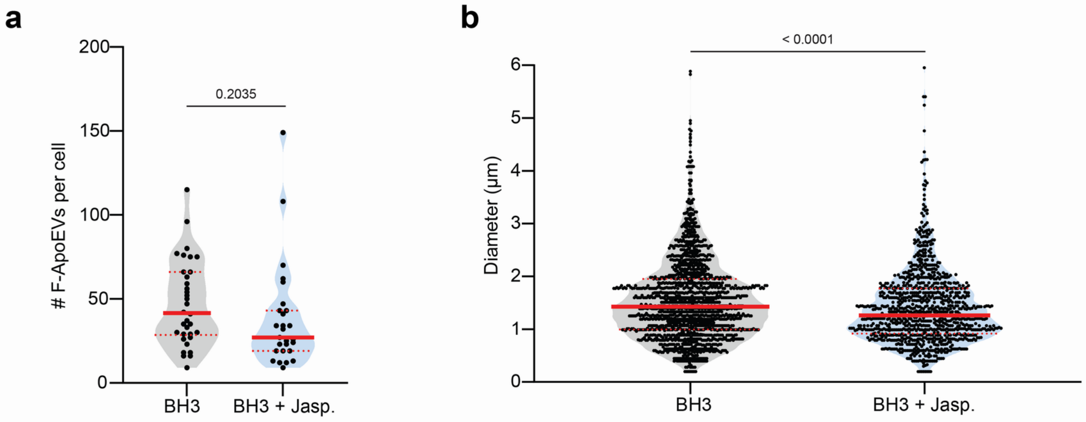

Supplementary Figure 9: Pharmacological inhibition of cell migration by jasplakinolide does not prevent the formation of FOOD. **a** Quantification of the number of F-ApoEVs generated per cell from confocal laser scanning microscopy (CLSM) imaging of apoptotic MEFs treated with a BH3-mimetic cocktail (5 µM ABT-737, 10 µM S63845) alone, or with migration inhibitor Jasplakinolide (50 nM). Data points represent individual cells (n=34 and n=27, respectively) pooled from (n=3) independent experiments. **b** Quantification of the diameter (µm) of F-ApoEVs from CLSM imaging of apoptotic MEFs with and without Jasplakinolide treatment (50 nM). Data points represent individual F-ApoEVs (n=1625 and n=1014) pooled from (n=3) independent experiments. Solid red line indicated mean, dashed red line indicates quarterlies. Unpaired student's two tailed t-test was performed to determine the indicated p-values.

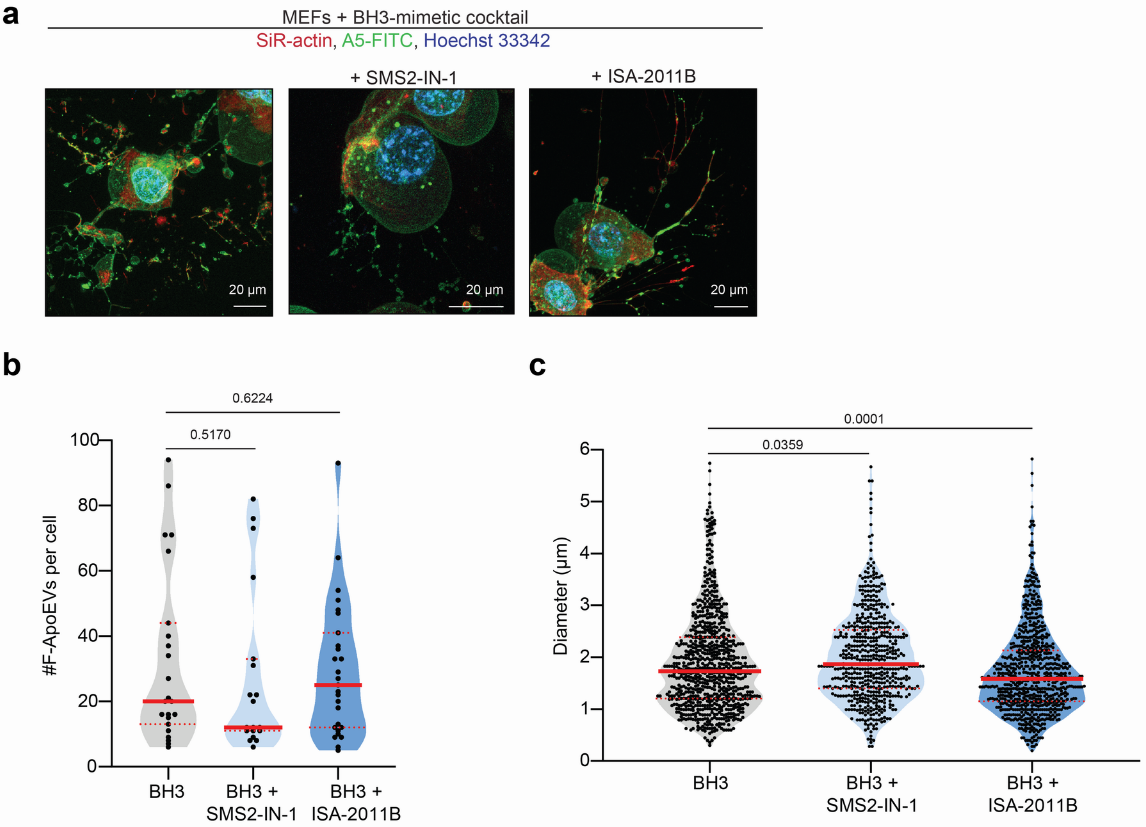

Supplementary Figure 10: Pharmacological inhibitors of migrasome formation do not inhibit F-ApoEV formation. a Confocal laser scanning microscopy (CLSM) imaging of apoptotic MEFs treated with a BH3-mimetic cocktail (5 µM ABT-737, 10 µM S63845) with and without migrasome inhibitors SMS2-IN-1 (30 µM) and, ISA-2011B (20 µM). MEFs were stained with SiR-actin, A5-FITC, and Hoechst 33342, to visualise F-actin, the nucleus, and exposed phosphatidylserine, respectively. Quantification of the number of F-ApoEVs generated per cell (**b**), and the diameter of F-ApoEVs (µm) (**c**) generated by apoptotic MEFs alone, or in the presence of migrasome inhibitor SMS2-IN-1(30 µM), or ISA-2011B (20 µM). Data points in (**b**) represent individual cells (n=23, n=19, and n=27, respectively) pooled from (n=3) independent experiments. Data points in (**c**) represent individual F-ApoEVs (n=789, n=562 and n=750) pooled from (n=3) independent experiments. Solid red line indicated mean, dashed red line indicates quarterlies. Unpaired student's two tailed t-test was performed to determine the indicated p-values.

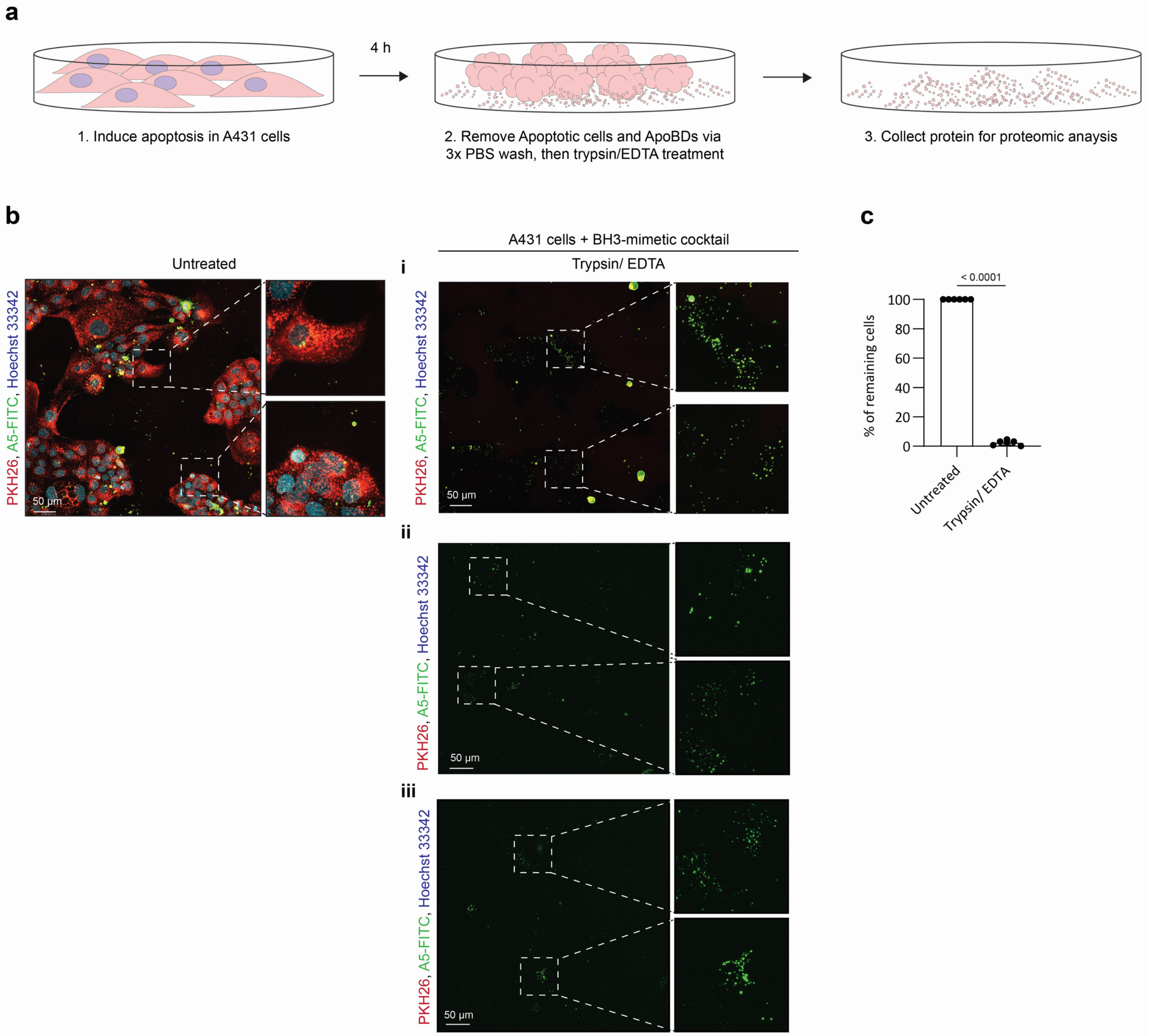

Supplementary Figure 11: In situ isolation of FOOD. a Schematic diagram of in situ FOOD isolation approach. **b** Representative confocal laser scanning microscopy images of untreated cells and FOOD post- treatment with trypsin/EDTA. **i**, **ii**, and **iii** indicate data generated from three independent experiments. **c** Quantification of remaining cells post FOOD isolation, determined by the number of cells remaining after isolation divided by the number of cells present in the untreated sample (n=6). Cells were stained with PKH26, and A5-FITC and Hoechst 33342. Error bars represent s.e.m. Unpaired student's two tailed t-test was performed to determine the indicated p values.

a

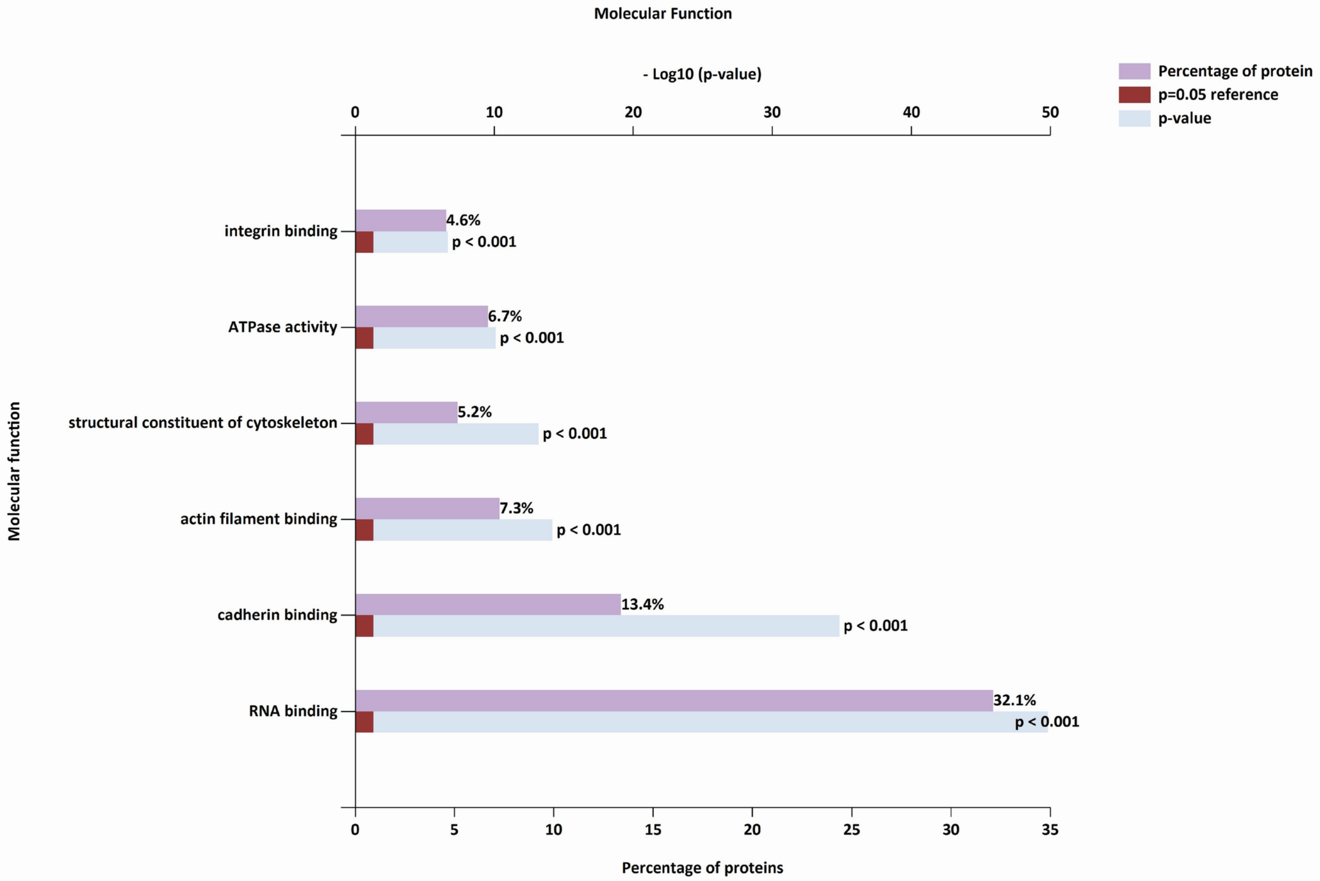

b

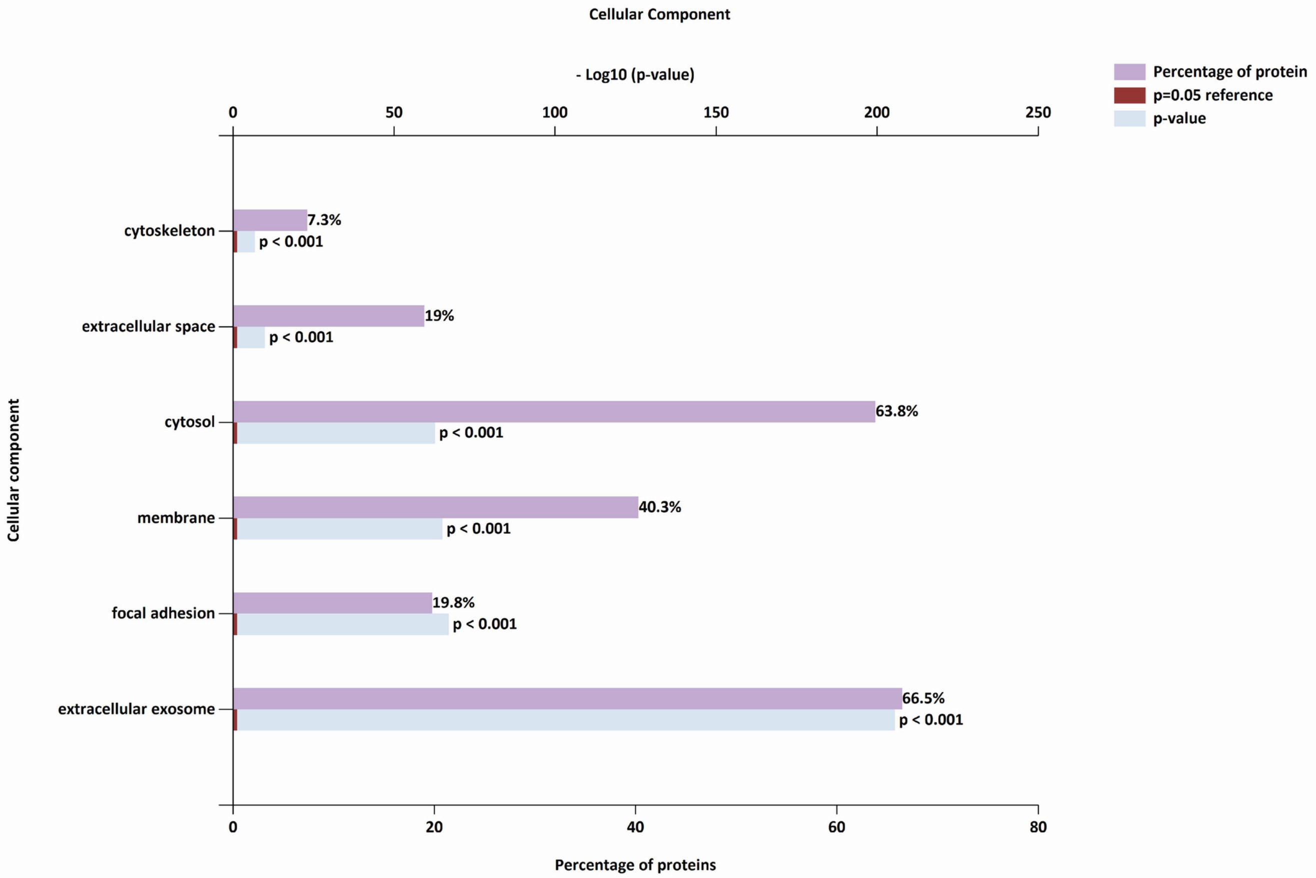

Supplementary Figure 12: Gene ontology enrichment analysis of FOOD/F-ApoEV proteins.
Gene ontology enrichment analysis of proteins isolated from FOOD/F ApoEVs according to (**a**) molecular function, and (**b**) cellular component, determined using Funrich software. Statistical significance were calculated by hypergeometric uncorrected p-value as determined by Funrich[73] software

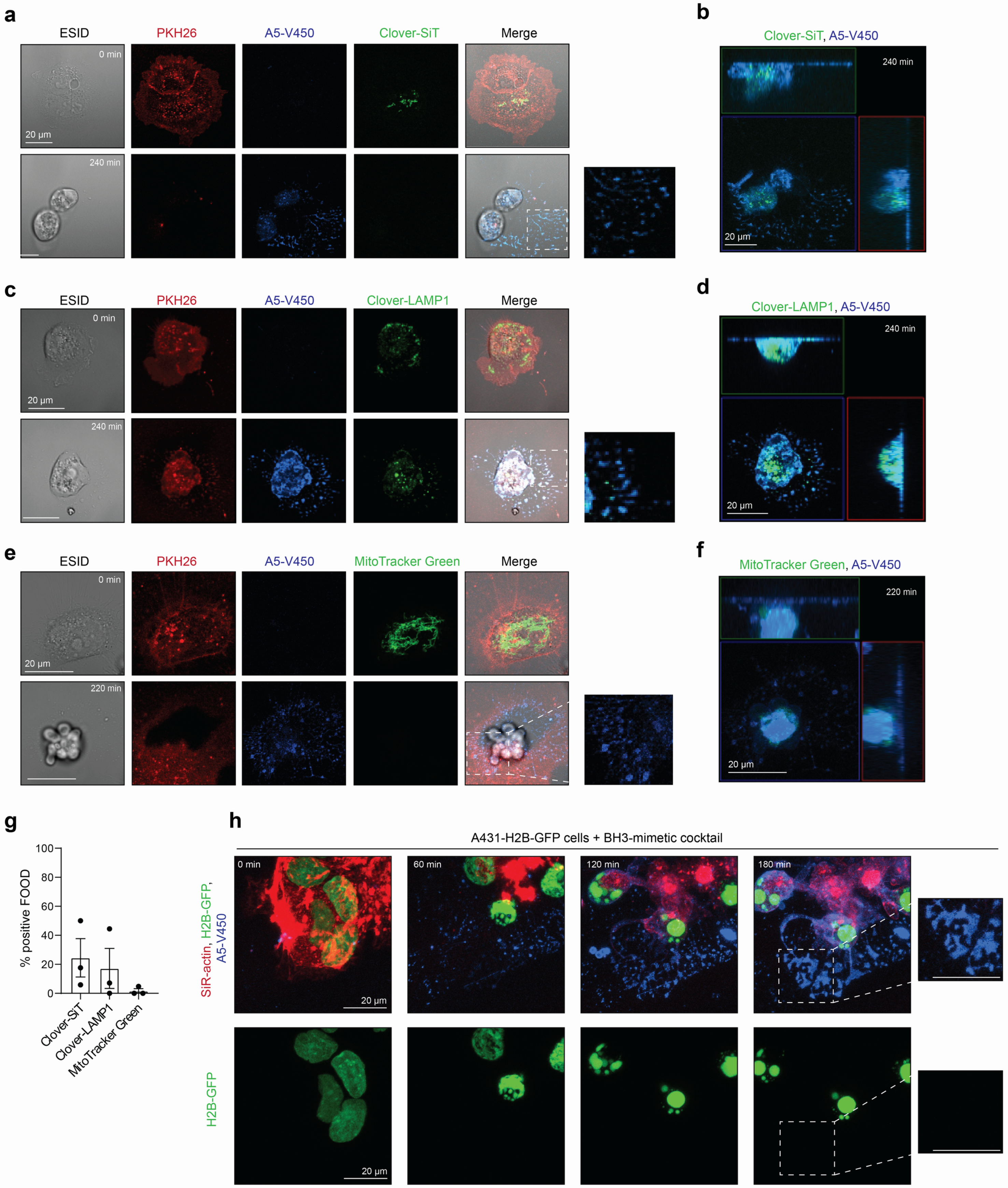

Supplementary Figure 13: Organelles and histones are not detected in FOOD. Representative confocal laser scanning micrscopy (CLSM) images of A431 cells following treatment with a BH3-mimetic cocktail (2.5 µM ABT-737, 0.5 µM S63845). Cells are expressing Clover-SiT (green) (**a**,**b**) Clover-LAMP1 (green) (**c**,**d**) or cells are stained with MitoTracker Green (**e**,**f**). Cells were stained with PKH26, and A5-V450. Enlarged images are shown right, with region of interest indicated by dotted square. Figures (**b**), (**d**), (**f**) and (**h**) all show MIP. **g** Quantification of the percentage of FOOD patches positive for each organelle. Error bars represent s.e.m. **h** Representative time-lapse CLSM images of A431 cells expressing H2B GFP (green) following treatment with a BH3-mimetic cocktail. Cells were additionally stained with SiR-actin, and A5-V450.

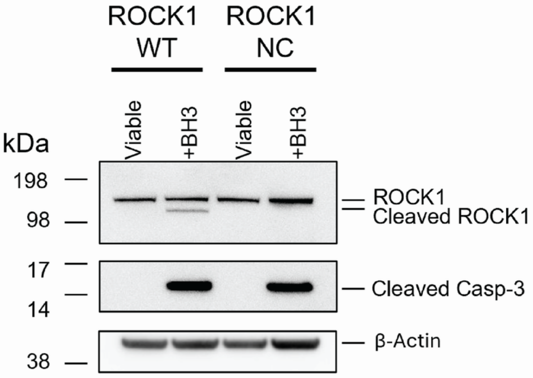

Supplementary Figure 14: ROCK1nc MEFs express a mutated form of ROCK1 resistant to caspase mediated cleavage. Immunoblot showing ROCK1nc MEFs express a mutated form of ROCK1 resistant to caspase mediated cleavage.

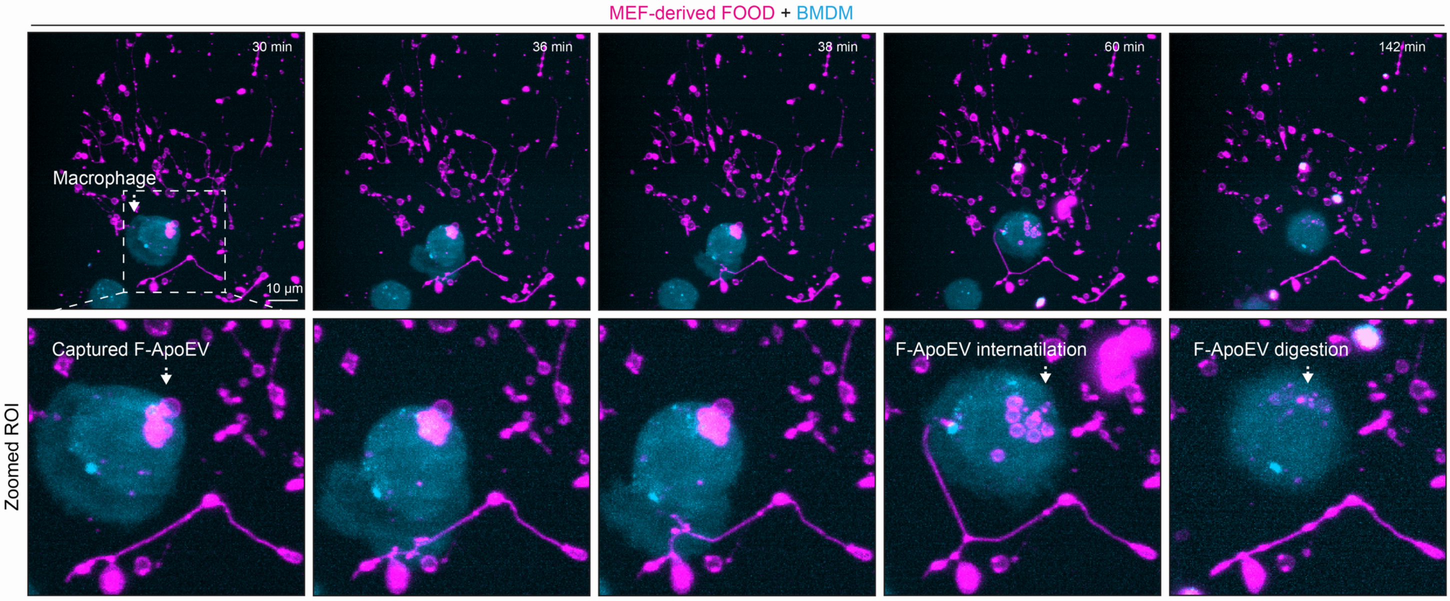

Supplementary Figure 15: Extended data: BMDM interaction with FOOD/F ApoEVs. Representative maximum intensity projection (MIP) images from time-lapse lattice light sheet microscopy (LLSM) of cell trace violet stained BMDM interacting with, engulfing, and digesting MEF-derived FOOD/ F-ApoEVs stained with A5-PE (magenta). Pannel below is zoomed region of above, as indicated by dotted box. Data is representative of (n=3) independent experiments.

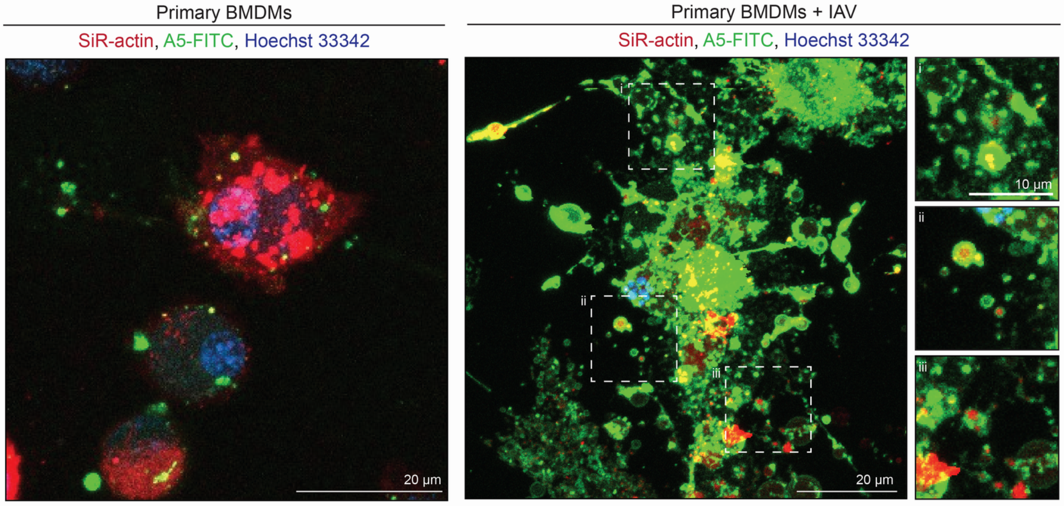

Supplementary Figure 16: FOOD/ F-ApoEV formation occurs in BMDMs infected with IAV. Representative maximum intensity projection images of FOOD/ F-ApoEV formation in BMDMs, untreated or IAV (24 h p.i.) (MOI=10). Cells were stained with SiR-actin, A5-FITC, and Hoechst 33342. Data is representative of (n=3) independent experiments.

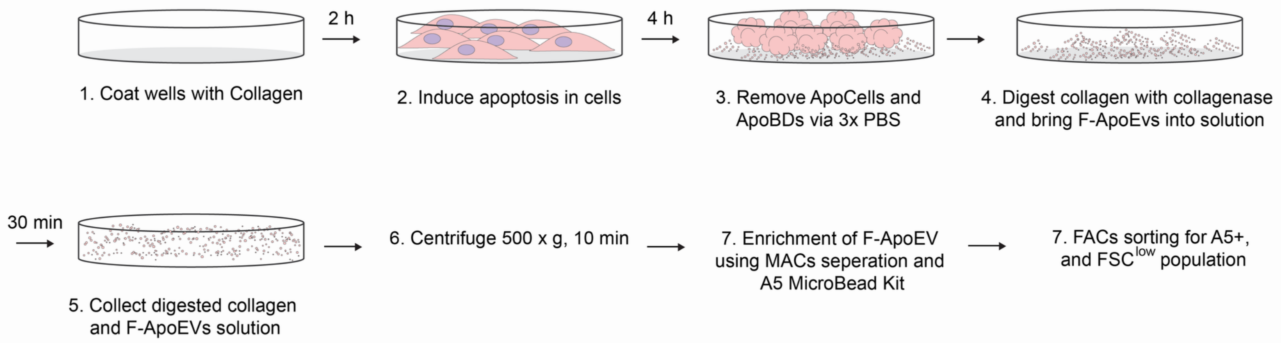

Supplementary Figure 17: Isolation schematic of F ApoEVs. Schematic diagram of isolation approach of F-ApoEVs from collagen coated tissue culture plates.

Supplementary References

72. Murray, M. B., Leak, L. B., Lee, W. C. & Dixon, S. J. Protocol for detection of ferroptosis in cultured cells. *STAR Protoc*. **4**, 102457 (2023).
