# Supplementary material for: The formation of the ‘footprint of death’ as a mechanism for generating large substrate-bound extracellular vesicles that mark the site of cell death
Source: Nat Commun. 2025 Oct 15;16:9160. doi: 10.1038/s41467-025-64206-3 (PMC12528690; doi:10.1038/s41467-025-64206-3)
Supplement: Supplementary file 2 — Description of Additional Supplementary File [file 41467_2025_64206_MOESM2_ESM.pdf]

Description of Additional Supplementary Files

File Name: Supplementary Video 1

Description: Time-lapse CLSM of FOOD formation in A431 epidermal cells treated with a BH3- mimetic cocktail (2.5 µM ABT-737, 0.5 µM S63845). Cell membrane and nucleus were visualised by PHK26 (red) and Hoechst 33342 (blue) staining, respectively, and exposed PtdSer was examined using A5-FITC (green). Data presented as MIP.

File Name: Supplementary Video 2:

Description: Time-lapse lattice light sheet microscopy (LLSM) images demonstrating rounding of FOOD into F-ApoEVs. Cells were stained with A5-FITC (blue) and TO-PRO-3 (magenta). Data is presented as a 3D MIP.

File Name: Supplementary Video 3:

Description: Time-lapse lattice light sheet microscopy (LLSM) images demonstrating FOOD BMDM interaction. BMDMs were stained with CTV (blue) and MEF-derived FOOD/ FApoEVs stained with A5-PE (magenta). Data is presented as a 3D MIP.

File Name: Supplementary Video 4:

Description: Time-lapse lattice light sheet microscopy (LLSM) images demonstrating FOOD BMDM engulfment. BMDMs were stained with CTV (blue) and MEF-derived FOOD/ FApoEVs stained with A5-PE (magenta). Data is presented as a 3D MIP.

File Name: Supplementary Data 1:

Description: Excel spreadsheet containing the list of proteins from A431-cell derived FOOD and apoptotic A431 cells.
