# Supplementary material for: The formation of the ‘footprint of death’ as a mechanism for generating large substrate-bound extracellular vesicles that mark the site of cell death
Source: Nat Commun. 2025 Oct 15;16:9160. doi: 10.1038/s41467-025-64206-3 (PMC12528690; doi:10.1038/s41467-025-64206-3)
Supplement: Supplementary file 8 — Reporting summary [file 41467_2025_64206_MOESM8_ESM.pdf]

nature portfolio

Corresponding author(s): Ivan K H Poon, Georgia K Atkin-Smith

Last updated by author(s): Jun 25, 2025

Reporting Summary

Nature Portfolio wishes to improve the reproducibility of the work that we publish. This form provides structure for consistency and transparency in reporting. For further information on Nature Portfolio policies, see our Editorial Policies and the Editorial Policy Checklist.

Statistics

For all statistical analyses, confirm that the following items are present in the figure legend, table legend, main text, or Methods section.

n/a	Confirmed	
☐	☒	The exact sample size (*n*) for each experimental group/condition, given as a discrete number and unit of measurement
☐	☒	A statement on whether measurements were taken from distinct samples or whether the same sample was measured repeatedly
☐	☒	The statistical test(s) used AND whether they are one- or two-sided<br>*Only common tests should be described solely by name; describe more complex techniques in the Methods section.*
☒	☐	A description of all covariates tested
☐	☒	A description of any assumptions or corrections, such as tests of normality and adjustment for multiple comparisons
☐	☒	A full description of the statistical parameters including central tendency (e.g. means) or other basic estimates (e.g. regression coefficient) AND variation (e.g. standard deviation) or associated estimates of uncertainty (e.g. confidence intervals)
☐	☒	For null hypothesis testing, the test statistic (e.g. *F*, *t*, *r*) with confidence intervals, effect sizes, degrees of freedom and *P* value noted<br>*Give P values as exact values whenever suitable.*
☒	☐	For Bayesian analysis, information on the choice of priors and Markov chain Monte Carlo settings
☒	☐	For hierarchical and complex designs, identification of the appropriate level for tests and full reporting of outcomes
☒	☐	Estimates of effect sizes (e.g. Cohen's *d*, Pearson's *r*), indicating how they were calculated

Our web collection on statistics for biologists contains articles on many of the points above.

Software and code

Policy information about availability of computer code

Data collection	Zeiss ZEN 3.10, SynGene, Hitachi SU7000, AMT Capture Engine (v 7.01)
Data analysis	FlowJo v10.8.2, Zeiss ZEN 3.10, Fiji (v 2.2.0), Gen5 software v 3.05, MaxQuant (v 1.6.17.0), Funrich (v 3.1.3), Excel (v 2405)

For manuscripts utilizing custom algorithms or software that are central to the research but not yet described in published literature, software must be made available to editors and reviewers. We strongly encourage code deposition in a community repository (e.g. GitHub). See the Nature Portfolio guidelines for submitting code & software for further information.

Data

Policy information about availability of data

All manuscripts must include a data availability statement. This statement should provide the following information, where applicable:

- Accession codes, unique identifiers, or web links for publicly available datasets
- A description of any restrictions on data availability
- For clinical datasets or third party data, please ensure that the statement adheres to our policy

All data presented in this study is original and not published elsewhere. Additional information is available upon request to the corresponding authors.

Research involving human participants, their data, or biological material

Policy information about studies with human participants or human data. See also policy information about sex, gender (identity/presentation), and sexual orientation and race, ethnicity and racism.

Reporting on sex and gender	N/A
Reporting on race, ethnicity, or other socially relevant groupings	N/A
Population characteristics	N/A
Recruitment	N/A
Ethics oversight	N/A

Note that full information on the approval of the study protocol must also be provided in the manuscript.

Field-specific reporting

Please select the one below that is the best fit for your research. If you are not sure, read the appropriate sections before making your selection.

☒ Life sciences ☐ Behavioural & social sciences ☐ Ecological, evolutionary & environmental sciences

For a reference copy of the document with all sections, see nature.com/documents/nr-reporting-summary-flat.pdf

Life sciences study design

All studies must disclose on these points even when the disclosure is negative.

Sample size	No statistical methods were used to predetermine sample size. To determine statistical significance of data, three or more biological repeats were were performed for all experiments.
Data exclusions	No data was excluded from the analyses.
Replication	Independent experiments were repeated at least three times, with all attempts of replication being successful.
Randomization	Randomization was not performed on in vitro experiments.
Blinding	Blinding was not required for data collection and analysis. To eliminate bias, all samples and control groups were prepared and analyzed simultaneously under identical conditions.

Reporting for specific materials, systems and methods

We require information from authors about some types of materials, experimental systems and methods used in many studies. Here, indicate whether each material, system or method listed is relevant to your study. If you are not sure if a list item applies to your research, read the appropriate section before selecting a response.

Materials & experimental systems

n/a	Involved in the study	
☐	☒	Antibodies
☐	☒	Eukaryotic cell lines
☒	☐	Palaeontology and archaeology
☒	☐	Animals and other organisms
☒	☐	Clinical data
☒	☐	Dual use research of concern
☒	☐	Plants

Methods

n/a	Involved in the study	
☒	☐	ChIP-seq
☐	☒	Flow cytometry
☒	☐	MRI-based neuroimaging

Antibodies

Antibodies used	Anti-Vinculin (hVIN-1) antibody (cat# v9264), rabbit anti-ROCK1 (Santa Cruz), rabbit anti-cleaved caspase 3 (Cell Signalling Technology), rabbit anti-PANX1 (Santa Cruz), anti-β actin (AC-15) antibody (Novus Biologicals; NB600-501), anti-rabbit-HRP (Abcam),

Sheep anti-mouse HRP (Cytiva) .

Validation: Antibodies were validated by the manufacturers and cited in literature as detailed in the product technical data sheet found on each manufacturer's website.

Eukaryotic cell lines

Policy information about cell lines and Sex and Gender in Research

Cell line source(s)	Human A431 cells, HeLa cells, Jurkat T cells and A549 cells were acquired from ATCC. Human umbilical vein endothelial cells (HUVEC) were acquired from Lonza. Mouse embryonic fibroblasts (MEFs) and mouse bone marrow derived macrophages (BMDMs) were generated in house.
Authentication	HeLa were authenticated using short tandem repeat (STR) profiling. MEFs were authenticated by immunoblot analysis (e.g. for ROCK1nc). Jurkat T cells and HUVEC were authenticated by cell surface marker analysis using flow cytometry [https://www.nature.com/articles/s41598-017-14305-z]. A431 and A549 cells were not authenticated.
Mycoplasma contamination	Mycoplasma testing was performed routinely and no mycoplasma contamination was detected.
Commonly misidentified lines (See ICLAC register)	No commonly misidentified cell lines were used in this study.

Plants

Seed stocks	N/A
Novel plant genotypes	N/A
Authentication	N/A

Flow Cytometry

Plots

Confirm that:

- [ ] The axis labels state the marker and fluorochrome used (e.g. CD4-FITC).
- [ ] The axis scales are clearly visible. Include numbers along axes only for bottom left plot of group (a 'group' is an analysis of identical markers).
- [ ] All plots are contour plots with outliers or pseudocolor plots.
- [ ] A numerical value for number of cells or percentage (with statistics) is provided.

Methodology

Sample preparation	For validation of apoptosis, cell sample was collected following trypsin treatment, then centrifuged (3000xg 5 min) to pellet. Harvested sample was resuspended into FACS buffer with 10% FCS and stained with various antibodies, or viability dyes, as appropiate.
Instrument	BD FACS CANTO II Flow Cytometer, BD Symphony A3 Flow Cytometer, LSRFortessa
Software	BD FACSDiva
Cell population abundance	For flow cytometry analysis, more than 5x10^5 events were acquired for analysis.
Gating strategy	Positive and negative cell populations (including apoptotic vesicles) were gated as per previous studies [https://www.nature.com/articles/s42003-020-0955-8; https://www.nature.com/articles/nprot.2016.028; https://www.nature.com/articles/s41418-019-0342-5; https://www.nature.com/articles/srep39846].

- [ ] Tick this box to confirm that a figure exemplifying the gating strategy is provided in the Supplementary Information.
